# Effect of Baicalin on Bacterial Secondary Infection and Inflammation Caused by H9N2 AIV Infection in Chickens

**DOI:** 10.1155/2020/2524314

**Published:** 2020-11-18

**Authors:** Xinheng Zhang, Qiqi Zhao, Xiaotong Ci, Sheng Chen, Liyi Chen, Jiamin Lian, Zi Xie, Yaqiong Ye, Huiyuan Lv, Hongxin Li, Wencheng Lin, Huanmin Zhang, Qingmei Xie

**Affiliations:** ^1^Lingnan Guangdong Laboratory of Modern Agriculture & Guangdong Provincial Key Lab of Agro-Animal Genomics and Molecular Breeding, College of Animal Science, South China Agricultural University, Guangzhou 510642, China; ^2^Guangdong Engineering Research Center for Vector Vaccine of Animal Virus, Guangzhou 510642, China; ^3^South China Collaborative Innovation Center for Poultry Disease Control and Product Safety, Guangzhou 510642, China; ^4^College of Life Science and Engineering, Foshan University, Foshan 528231, China; ^5^Beijing Center Biology CO., LTD., Beijing 100000, China; ^6^USDA, Agriculture Research Service, Avian Disease and Oncology Laboratory, East Lansing, MI 48823, USA

## Abstract

H9N2 subtype avian influenza virus (H9N2 AIV) is a low pathogenic virus that is widely prevalent all over the world. H9N2 AIV causes immunosuppression in the host and often leads to high rates of mortality due to secondary infection with Escherichia. Due to the drug resistance of bacteria, many antibiotics are not effective in the treatment of secondary bacterial infection. Therefore, the purpose of this study is to find effective nonantibiotic drugs for the treatment of H9N2 AIV infection-induced secondary bacterial infection and inflammation. This study proves, for the first time, that baicalin, a Chinese herbal medicine, can regulate *Lactobacillus* to replace *Escherichia* induced by H9N2 AIV, so as to resolve the intestinal flora disorder. In addition, baicalin can effectively prevent intestinal bacterial translocation of SPF chickens' post-H9N2 AIV infection, thus inhibiting secondary bacterial infection. Furthermore, baicalin can effectively treat H9N2 AIV-induced inflammation by inhibiting intestinal structural damage, inhibiting damage to ileal mucus layer construction and tight junctions, improving antioxidant capacity, affecting blood biochemical indexes, and inhibiting the production of inflammatory cytokines. Taken together, these results provide a new theoretical basis for clinical prevention and control of H9N2 AIV infection-induced secondary bacterial infection and inflammation.

## 1. Introduction

H9N2 avian influenza virus (AIV) is a kind of negative strand RNA virus that belongs to the influenza virus A genus of the *Orthomyxoviridae* family [1]. H9N2 avian influenza virus (AIV) is a low-pathogenic virus that is found in many bird and poultry species throughout the world [2]. H9N2 can replicate not only in the respiratory epithelia but also in the gastrointestinal tract [3, 4]. Although H9N2 has low pathogenicity, its infection often leads to secondary infection with avian pathogens, which can increase the viral titer in tissues as well as the severity of clinical signs and can lead to a high mortality rate in infected birds, hence causing serious economic losses to the poultry industry [5]. H9N2 AIV can be isolated from host tissues including the trachea, lung, brain, spleen, pancreas, cloacal cavity, and intestinal tract, and it causes inflammation and enteric problems to hosts [6]. H9N2 AIV can be used as a gene donor that mutates into other subtypes of viruses, such as the highly pathogenic H7N9 and H10N8, thereby infecting humans and mammals. Therefore, H9N2 poses a serious threat to the aquaculture industry and human health and security [7, 8].

The commensal gut microbiota has been reported to play a key role in viral pathogenesis by regulating the immune response against influenza virus [9]. However, on the other hand, intestinal microbe disorders can increase the severity of disease [10]. The balance of gut microecology is an important factor in the body's resistance to disease caused by influenza virus [9]. Recently, we reported that H9N2 AIV infection produces inflammation of the mucosal epithelium, which leads to secondary bacterial infection due to the invasion of *Escherichia coli* [11]. Many clinical studies have also shown that H9N2 AIV is highly susceptible to secondary infections of bacteria (especially *Escherichia coli*) and other pathogens, resulting in high rates of mortality in poultry [12, 13]. The occurrence of inflammation usually affects oxidative stress and blood biochemical indexes, and intestinal inflammation will lead to intestinal tissue damage, intestinal mucus layer structure and tight junction damage, and the induction of inflammatory cytokine production [11, 14, 15]. In the breeding industry, antibiotics are usually used to treat H9N2 AIV-induced inflammation, but extensive use will lead to the emergence of drug-resistant strains, and drug residues in livestock and poultry products not only cause environmental pollution but also pose a threat to human health.

Baicalin is a flavonoid compound purified from the medicinal plant *Scutellaria baicalensis* Georgi that is widely used as a traditional Chinese herbal medicine for its anti-inflammatory activities [16, 17]. Baicalin has been used to treat a variety of inflammatory diseases such as bronchitis, hepatitis, nephritis, and atopic dermatitis [18]. In addition, baicalin has strong effects against dengue virus, hepatitis B virus, and enterovirus 71 [19]. Baicalin has been reported to be effective against H1N1/H3N2 influenza A virus in both cell culture and a mouse model [20]. However, whether baicalin is effective in treating H9N2 AIV infection-induced secondary bacterial infection and inflammation has not been reported. Therefore, the purpose of this study is to determine whether baicalin can effectively inhibit H9N2 AIV-induced secondary bacterial infection and inflammation in chickens.

## 2. Materials and Methods

### 2.1. Ethics Statement

The use of animals in this study was approved by the Animal Care Committee of South China Agricultural University (approval ID: SYXK-2014-0136).

### 2.2. Viral, Baicalin, Chickens

The H9N2 subtype avian influenza strain A/Chicken/Henan/SH01/2015 (SH0115) (GenBank No. KT023065) was preserved in our laboratory and was used in all related experiments that can cause high morbidity and mortality due to diarrhea and secondary bacterial infections such as *Escherichia coli* [11]. Baicalin was purchased from Beijing Shengtaier Technology Co., Ltd. The purity of baicalin was 85%, and other components were total flavonoids. One hundred and twenty-eight 1-day-old healthy SPF chickens with basically the same body weight were purchased from the SPF Experimental Animal Center of Guangdong Dahuanong Animal Health Co., Ltd.

### 2.3. Experimental Design

Among the 128 1-day-old SPF chickens, 80 1-day-old SPF chickens were randomly divided into 4 groups with 20 chickens per group: a mock group, an H9N2 infection group, a baicalin-fed group, and an H9N2 AIV infection plus baicalin-fed group. They were fed in a negative pressure isolator. The specific experimental groups are shown in Table 1. Routine feeding and management were carried out with food and drinking water freely available. The additive amount of baicalin was 0.5 g baicalin powder per 1000 g feed. We began to feed baicalin to the corresponding groups at the age of 1 day. The composition and nutritional level of basal feeding were shown in Table 2 [21]. The chickens in the H9N2 AIV infection group and H9N2 AIV infection plus feeding baicalin group were infected with H9N2 AIV at the age of 9 days, and the infection dose was 3-fold 10^6^ EID_50_/0.1 mL. H9N2 AIV infection was carried out by nasal drip. At the same time of viral infection, 0.1 mL phosphate buffer saline (PBS) was dripped into nasal cavity of chickens in mock group and feeding baicalin group. Those 80 1-day-old SPF chickens were used for the following study: 16s rRNA high-throughput sequencing of intestinal microbiota, isolation of microflora from visceral organs, histological examination of intestinal segments and villus conditions, detection of antioxidant and blood biochemical indexes, and detection of intestinal immune-related cytokines, ileum mucus layer structure, and tight junction-related factors by quantitative real-time PCR (RT-qPCR).

The other 48 1-day-old SPF chickens were used for the study of Neongreen-tagged bacteria isolation. Those 48 1-day-old healthy SPF chickens were divided into four groups: a mock group, an H9N2 AIV infection group, a baicalin group, and an H9N2 AIV infection with baicalin-feeding group. Baicalin was introduced to the corresponding groups at the age of 1 day. The additive amount of baicalin was 0.5 g baicalin powder per 1000 g feed. At the age of 9 days, the infection dose of H9N2 virus was 3-fold 10^6^ TCID_50_/0.1 mL, which lasted for three days. On the third day after virus infection, all chickens were fed with Neongreen bacteria at a dose of 300 *μ*L 1 × 10^9^ CFU/mL per chicken.

### 2.4. 16s rRNA High-Throughput Sequencing of Intestinal Microbiota

Three chickens were randomly selected from the mock group, H9N2 infection group, baicalin-fed group, and H9N2 AIV infection plus baicalin-fed groups, respectively, at 5 days and 12 days postinfection (dpi) with H9N2 AIV. After carotid artery bloodletting, the contents of the ileum were taken and placed in an aseptic freezer under aseptic conditions. Using the FastDNA SPIN Kit for Soil kit, the total DNA of the ileal contents of the chicken was extracted according to the steps described in the manufacturer, and the purity, concentration, and integrity of the extracted DNA were detected by NanoDrop 2000 spectrophotometer (ThermoFisher Scientific Co., Waltham, MA, USA) using the 260/280 nm absorbance ratio and 1% agarose gel electrophoresis. In order to ensure the accuracy and reliability of the follow-up data analysis, the V3–V4 variable regions of 16s rRNA of the qualified samples were amplified by PCR. After the Illumina Miseq PE300 sequencing was completed in the sequencing platform, the effective sequences were filtered and optimized, and OTUs (Operational Taxonomic Units) were defined to analyze the biological information of all sequences according to the similarity of more than 97% sequences.

### 2.5. Isolation of Microflora from Visceral Organs

Three chickens were randomly selected from the mock group, H9N2 infection group, baicalin-fed group, and H9N2 AIV infection plus baicalin-fed group, respectively, at 5 days and 12 days postinfection (dpi) with H9N2 AIV. After carotid artery bloodletting, livers, lungs, and mesentery were obtained under aseptic conditions. An appropriate amount of corresponding tissue was put into the grinding tube with 1 mL normal saline. After each tissue was fully homogenized, the mixture of 200 *μ*L homogenate in each tube was evenly applied on the prepared LB plate. After the treated plates were cultured in a constant temperature incubator at 37°C for 12 hours, the growth of bacteria on each plate was recorded statistically, and those with colonies were recorded as positive, and those without colonies were negative.

### 2.6. Neongreen-Tagged Bacteria Isolation

Slaughtering samples were taken at 12, 24, 36, and 48 hours after the administration of Neongreen bacteria in the mock group, H9N2 infection group, baicalin-fed group, and H9N2 AIV infection plus baicalin-fed group, respectively. Neongreen-tagged bacteria was the Neongreen *E. coli* bacteria modified by the NeonGreen fluorescence labeling gene. Aseptic collection of a mixture of the inner wall and lumen of the ileum, liver, lungs, and mesentery was conducted. The homogenate was homogenized in a sterile homogenate tube containing 1 mL saline. One hundred microliters of homogenate mixture was taken to the LB plate with ampicillin resistance, and the bacterial solution was coated and cultured in a 37°C incubator for 12 hours. When bacterial growth on the plate was observed, the colony growth was judged to be positive, while noncolony growth was judged to be negative.

### 2.7. Histological Examination of Intestinal Segments and Villus Conditions

Three chickens were randomly selected for the mock group, H9N2 infection group, baicalin-fed group, and H9N2 AIV infection plus baicalin-fed group, respectively, at 5 days and 12 days postinfection (dpi) with H9N2 AIV. After carotid artery bloodletting, the ileum and cecum were fixed in formalin and made into HE staining sections. Intestinal sections were observed with a Nikon Eclipse ci optical microscope and analyzed and photographed with the NIS-Elements F 4.00.00 software (Nikon Instruments, Inc., Melville, NY, USA). Three intact intestinal villi were selected for each section. The height and corresponding crypt depth of selected intestinal villi were measured, the villus height to crypt depth ratio was calculated, and the average value was taken as the final measured value.

### 2.8. Detection of Antioxidant and Blood Biochemical Indexes

Three chickens were randomly selected from the mock group, H9N2 infection group, baicalin-fed group, and H9N2 AIV infection plus baicalin-fed group, respectively, at 5 days and 12 days postinfection (dpi) with H9N2 AIV. Two milliliters of blood was collected from the aseptic vein under the wing. The blood was placed at room temperature without anticoagulant treatment, and the serum was isolated by 2143 g centrifugation for 10 minutes after agglutination. The kits used for the determination of total antioxidant capacity, glutathione peroxidase, superoxide dismutase, and malondialdehyde were purchased from the Nanjing Jiancheng company (Nanjing, China). The procedures were performed according to the instructions in the different kits. Serum urea nitrogen, low-density cholesterol, high-density cholesterol, aspartate aminotransferase, total cholesterol, and alanine aminotransferase concentrations were measured by a Mindray BS-5800M automatic biochemical analyzer (Shenzhen, China).

### 2.9. Detection of Intestinal Immune-Related Cytokines, Ileum Mucus Layer Structure, and Tight Junction-Related Factors by Quantitative Real-Time PCR (RT-qPCR)

Three chickens were randomly selected from the mock group, H9N2 infection group, baicalin-fed group, and H9N2 AIV infection plus baicalin-fed group, respectively, at 5 days and 12 days postinfection (dpi) with H9N2 AIV. Under an aseptic environment, a segment of the ileum was cut off and put into a 2 mL grinding tube containing 1 mL RNAiso. According to the sequences published in GenBank, primers for RT-qPCR detection of intestinal inflammation-related cytokines were designed and the related primer sequences are shown in Table 3. Primers for the ileum mucus layer structure and tight junction-related factors are listed in Table 4. Total RNA was extracted from the ileum epithelium of each broiler with TRIzol (Invitrogen, Carlsbad, CA, USA). The purity and concentration of the total RNA were measured in a NanoDrop 2000 spectrophotometer (ThermoFisher Scientific Co., Waltham, MA, USA) using the 260/280 nm absorbance ratio and then a 1.0 *μ*g RNA of each sample was reverse-transcribed into cDNA using the RecerTra Ace qPCR RT Master Mix with gDNA remover (TOYOBO Co., LTD. Life Science Department, OSAKA, Japan), and the cDNA was stored at -20°C. GAPDH was as the internal reference gene. The target gene was amplified by RT-qPCR, and the relative expression of each gene was calculated by 2^-△△Ct^ [11].

### 2.10. Statistics

Statistical analysis was performed using the SPSS 13.0 statistical software (SPSS, Inc.). The relative abundance analysis of microflora, intestinal structural damage analysis, ileal tight junction protein, antioxidant capacity analysis, blood biochemical indexes, and inflammatory cytokine analysis were analyzed by Student's *t*-tests, and then one-way analysis of variance (ANOVA) with the Tukey post hoc tests was used to compare the differences among different treatment groups. For each separate set of experiments, three independent biological replicates were evaluated. Six technical replicates were used for RT-qPCR. Data are presented as the mean ± standarddeviation.

## 3. Result

### 3.1. Baicalin Inhibited Intestinal Microflora Disorder of SPF Chickens' Post-H9N2 AIV Infection

**Figure 1:**
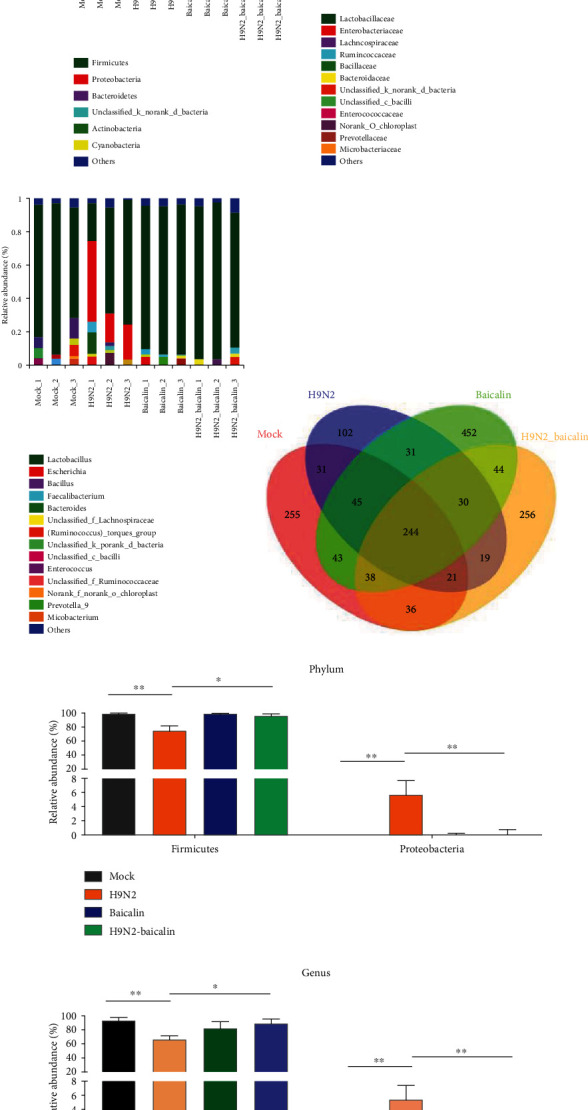
The addition of baicalin regulated the ileal microbiota composition caused by H9N2 AIV infection in chickens at 5 dpi and 12 dpi. The ileal microbiota from mock, H9N2 AIV infection, baicalin-fed, and H9N2 AIV infection plus baicalin groups at 5 dpi was analyzed by sequencing using the Illumina HiSeq system. The relative abundances of the (a) bacterial phyla, (b) families, and (c) genera are displayed. (d) A Venn diagram of OTUs (operational taxonomic units) in different groups at 5 dpi is shown. The ileal microbiota from the mock, H9N2 AIV infection, baicalin-fed, and H9N2 AIV infection and baicalin groups at 12 dpi was analyzed by sequencing using the Illumina HiSeq system. The relative abundances of the (e) bacterial phyla and (f) genera are displayed. The cutoff abundance level was set at 0.01%. Data are presented as the mean ± standarddeviation of three independent biological experiments. The differences between groups were analyzed using ANOVA. ^∗^*P* < 0.05 and ^∗∗^*P* < 0.01.

Firstly, we used cloacal swabs to detect whether the live virus was present in each infected group. All chickens in the infected group tested positive for H9N2 AIV. Then, the microbiota composition in the ileal contents of the mock group, H9N2 AIV infection group, baicalin-fed, and H9N2 infection with baicalin (H9N2-baicalin) groups was analyzed by 16S rRNA high-throughput sequencing. Firmicutes were the major bacterial phyla present in all samples from the different groups at 5 days postinfection (dpi) (Figure 1(a)). The proportion of *Lactobacillaceae* at 5 dpi significantly decreased in the H9N2 AIV infection group, while the quantity of *Enterobacteriaceae* significantly increased at the family level (Figure 1(b)). Furthermore, at 5 dpi, the proportion of *Lactobacillus* significantly decreased in the H9N2 AIV infection group, while the quantity of *Escherichia* significantly increased at the genus level (Figure 1(c)). These results, once again, indicated that H9N2 AIV infection can induce inflammation. However, the addition of baicalin could significantly inhibit the increases in *Proteobacteria*, *Enterobacteriaceae*, and *Escherichia* caused by H9N2 AIV infection and restore the concentrations of *Firmicutes*, *Lactobacillaceae*, and *Lactobacillus* in the intestine at the phylum, family, and genus levels at 5 dpi (Figures 1(a)–1(c)). The results show that the addition of baicalin could inhibit the disturbance of flora caused by H9N2 AIV infection at 5 dpi as well as restoring the original quantity of beneficial bacteria in the intestinal tract.

In addition, compared with the control group at 5 dpi, the number of OTUs increased and the number of specific OTUs increased by 214 and 197 in the baicalin-added group. The number of OTUs increased and the number of specific OTUs decreased by 190 and 153, respectively, in the H9N2 AIV infection group. Compared with the H9N2 AIV infection group, the number of OTUs and the number of specific OTUs in the H9N2 AIV infection with baicalin group increased by 165 and 154, respectively (Figure 1(d)). The results indicated that H9N2 AIV infection and the addition of baicalin can cause changes in the intestinal flora, resulting in their own specific flora patterns, and the addition of baicalin can produce more specific flora. The addition of baicalin will alleviate the decrease in the number of species of the original intestinal flora caused by H9N2 AIV infection and can produce more specific flora.

Similarity, the addition of baicalin could significantly inhibit the increases in *Proteobacteria* and *Escherichia* caused by H9N2 AIV infection and restore the concentrations of *Firmicutes* and *Lactobacillus* in the intestine at the phylum and genus levels, respectively, at 12 dpi (Figures 1(e) and 1(f)). These results indicated that the addition of baicalin could effectively inhibit the increase of *Escherichia* caused by H9N2 AIV infection at 5 dpi and 12 dpi and restore the quantity of beneficial bacteria in the intestinal tract.

### 3.2. Baicalin Effectively Prevented Intestinal Bacterial Translocation of SPF Chickens' Post-H9N2 AIV Infection

**Figure 2:**
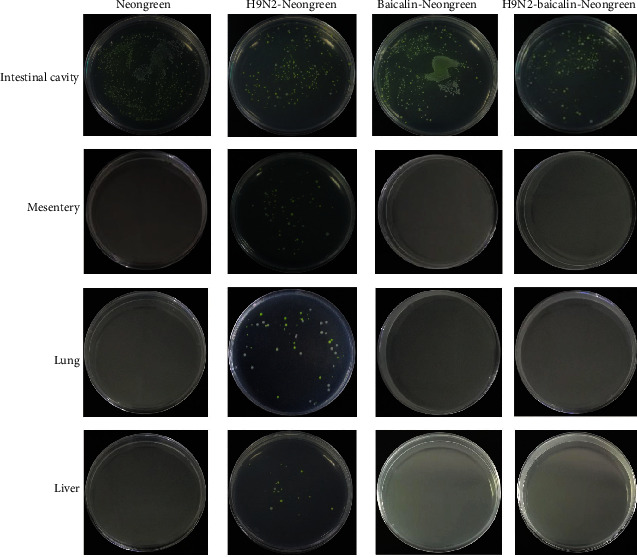
The addition of baicalin effectively prevented translocation of Neongreen-labeled bacteria in SPF chickens' post-H9N2 AIV infection. Isolation of Neongreen-labeled bacteria from the intestinal cavity, mesentery, lungs, and liver was conducted in different groups including Neongreen, H9N2-Neongreen, baicalin-Neongreen, and H9N2-baicalin-Neogreen after the infected chickens were drenched with labeled bacteria at 12, 24, 36, and 48 hours.

Studies have shown that H9N2 AIV infection can promote the translocation of intestinal flora in mice [22], but the translocation of microflora in chicken intestine caused by H9N2 AIV infection is still unknown. Our study shows that H9N2 infection could promote translocation of the microflora from the intestinal metastasis to the mesentery, liver, and lungs in chickens at 5 dpi and 12 dpi. However, no bacteria were isolated from the liver, lungs, or mesentery after feeding baicalin in the infection group **(**Table 5**)**. Furthermore, the Neongreen-labeled bacteria strain was used to trace the phenomenon of bacterial translocation. Our results show that the Neongreen-labeled bacteria were isolated from the intestinal cavity of all chickens subjected to H9N2 AIV infection followed by administration of Neongreen-labeled bacteria, while no Neongreen-labeled bacteria were isolated from the liver, lungs, or mesentery in the baicalin plus H9N2 AIV infection group at 12, 24, 36, and 48 hours **(**Table 6**)**. The translocation of Neongreen-labeled bacteria in chickens of different treatment groups is shown in Figure 2. These results show that baicalin treatment effectively prevented the Neongreen-labeled bacteria translocation caused by H9N2 AIV infection in SPF chickens, indicating that baicalin can effectively inhibit secondary bacterial infection caused by H9N2 AIV in chickens.

### 3.3. Baicalin Minimized Intestinal Structure Injury in SPF Chickens' Post-H9N2 AIV Infection

**Figure 3:**
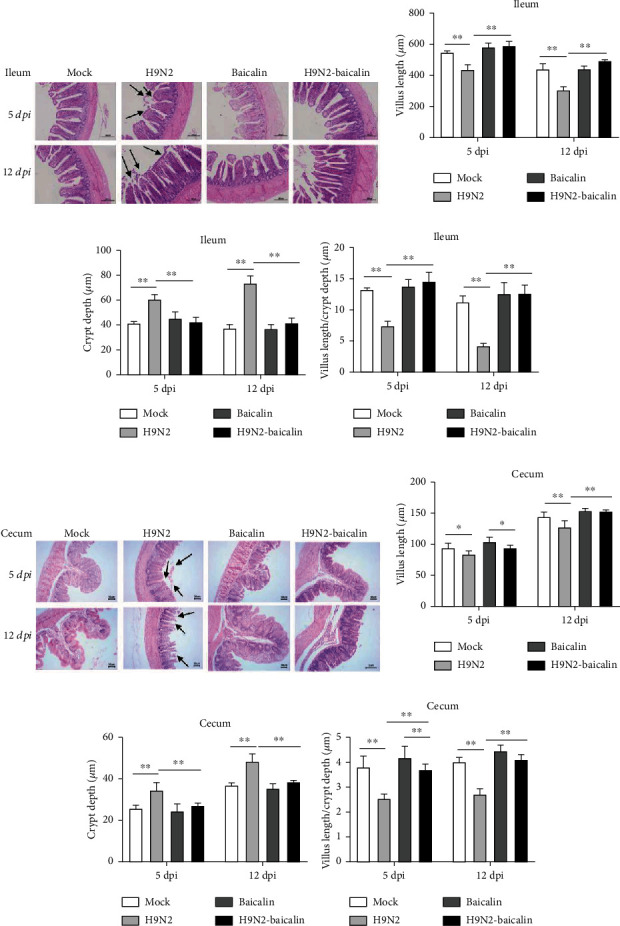
The addition of baicalin alleviated intestinal histopathological changes in the ileum and cecum caused by H9N2 AIV infection at 5 dpi and 12 dpi. (a) Histological features of the ileal mucosa were compared between the H9N2 AIV infection plus baicalin (H9N2-baicalin) and H9N2 AIV infection groups using hematoxylin and eosin staining at 5 dpi and 12 dpi. Images are provided at a lower magnification (100x) for histological observation and statistics. The (b) villus length (c) and crypt depth of the ileum were measured by Image-Pro Plus 6.0. (d) The spatial distribution of the villus length/crypt depth of the ileum is shown. (e) Histological features of cecum mucosa between the group of feeding baicalin with H9N2 AIV infection (H9N2-baicalin) and H9N2 AIV infection were investigated with hematoxylin and eosin staining at 5 dpi and 12 dpi. Images are provided at a lower magnification (100x) for histological observation and statistics. The (f) villus length and (g) crypt depth of the cecum were measured with the Image-Pro Plus 6.0 software. (h) The spatial distribution of villus length/crypt depth of the cecum is shown. Data are presented as the mean ± standarddeviation of three independent biological experiments. The differences between groups were analyzed using ANOVA. ^∗^*P* < 0.05 and ^∗∗^*P* < 0.01.

We used histopathological sections to analyze the intestinal structure injury caused by H9N2 AIV infection and verify whether baicalin could inhibit the injury. The ileac histopathology analysis showed that at 5 dpi, the mucosal epithelial cells were degenerated and exfoliated, the villi were damaged or even exfoliated, and the myometrium cells were damaged in the H9N2 AIV infection group. At 12 dpi, the myometrium was damaged in the H9N2 AIV infection group. However, there was no injury to the ileum in baicalin plus H9N2 AIV infection group at 5 dpi and 12 dpi, indicating that baicalin could minimize the occurrence of structural injury to the ileum caused by H9N2 AIV infection (Figure 3(a)). Similarly, at 5 dpi, the cecal mucosal epithelial cells were exfoliated and the myometrium was thinned obviously in the infected group. At 12 dpi, the mucosal epithelial cells of the cecum were exfoliated, the villi were destroyed, and the muscular tissue became thinner in the infected group. However, the cecal structure of the group fed with baicalin plus exposure to H9N2 AIV infection was the same as that of the group fed with baicalin alone (Figure 3(e)). These results indicated that baicalin could effectively relieve the pathological changes in the mucosal epithelial villi and minimize intestinal mucosal damage caused by H9N2 AIV infection.

The length of villi in the ileum and cecum significantly reduced in the H9N2 AIV infection group at 5 dpi and 12 dpi, while the addition of baicalin to the infected chickens significantly upregulated the length of villi in the ileum and cecum (*P* < 0.01 and *P* < 0.05) (Figures 3(b) and 3(f)). The crypt depth of the ileum and cecum significantly increased at 5 dpi and 12 dpi, while the addition of baicalin to the infected chickens significantly downregulated the crypt depth of the ileum and cecum (*P* < 0.01) (Figures 3(c) and 3(g)). The regular mucosal villus length/crypt depth of the ileum and cecum was significantly reduced in the H9N2 AIV infection group at 5 dpi and 12 dpi, while the addition of baicalin in the infected chickens significantly increased the villus length/crypt depth ratio of the ileum and cecum at 5 dpi and 12 dpi compared with the H9N2 AIV infection group (*P* < 0.01) (Figures 3(d) and 3(h)). These results indicated that the addition of baicalin minimized the occurrence of intestinal structural injury of the chickens' post-H9N2 AIV infection.

### 3.4. Baicalin Inhibited the Damage to Ileal Mucus Layer Construction and Tight Junctions of SPF Chickens' Post-H9N2 AIV Infection

**Figure 4:**
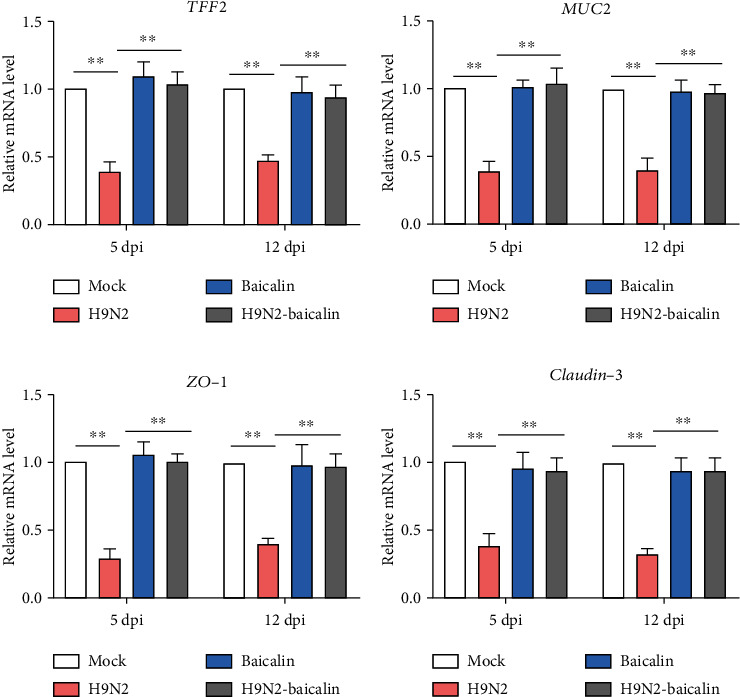
The addition of baicalin increased the mRNA expression levels of *TFF2*, *MUC2*, *ZO-1*, and *Claudin-3* that were downregulated by H9N2 AIV infection in the ileal epithelial cells, as found by RT-qPCR. (a) The mRNA expression level of *TFF2* at 5 dpi and 12 dpi. (b) The mRNA expression level of *MUC2* at 5 dpi and 12 dpi. (c) The mRNA expression level of *ZO-1* at 5 dpi and 12 dpi. (d) The mRNA expression level of *Claudin-3* at 5 dpi and 12 dpi. Data are presented as themean ± standarddeviation of three independent biological experiments. The differences between groups were analyzed using ANOVA. ^∗∗^*P* < 0.01.

In order to explore the damage to the ileal mucus layer construction and tight junctions of SPF chickens' post-H9N2 AIV infection, the mRNA expression of related genes was detected. TFF2 is a kind of gastrointestinal mucosal secretory protein. At 5 dpi and 12 dpi, the expression of TFF2 was significantly inhibited, while the addition of baicalin in the infected group significantly increased the mRNA expression of TFF2 (*P* < 0.01) (Figure 4(a)). The expression of MUC2, a protective and antimicrobial mucoprotein, was also significantly decreased in the H9N2 infection group at 5 dpi and 12 dpi, while the mRNA expression level of MUC2 in the baicalin plus H9N2 AIV infection group was significantly upregulated (*P* < 0.01) (Figure 4(b)). These results suggested that the addition of baicalin can protect the ileal mucus layer structure from damage caused by H9N2 infection in chickens. Next, we examined the genes associated with the tight junctions of mucosal epithelial cells such as cytoplasmic protein ZO-1 and cell membrane protein Claudin-3. The results show that the mRNA expression of the cytoplasmic protein ZO-1 and the cell membrane protein Claudin-3 significantly decreased at 5 dpi and 12 dpi, while the mRNA expression of Claudin-3 and ZO-1 significantly increased in the baicalin plus H9N2 AIV infection group (*P* < 0.01) (Figures 4(c) and 4(d)). These results indicate that baicalin can inhibit the damage done to the ileal mucus layer construction and tight junctions of the chickens' post-H9N2 AIV infection.

### 3.5. Baicalin Improved the Antioxidant Capacity of SPF Chickens' Post-H9N2 AIV Infection

**Figure 5:**
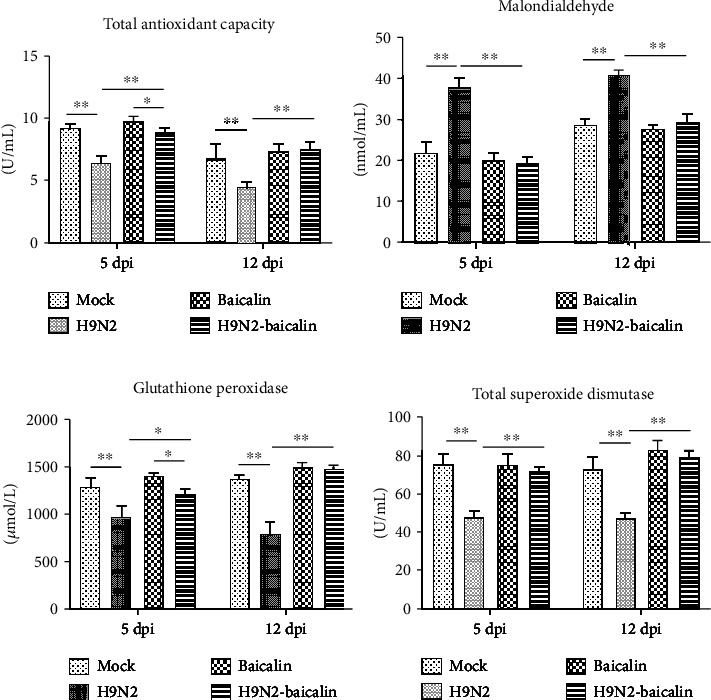
The addition of baicalin improved the antioxidant capacity of SPF chickens' post-H9N2 AIV infection. (a) Baicalin reversed the downregulation of the serum total antioxidant capacity caused by H9N2 AIV infection at 5 dpi and 12 dpi. (b) Baicalin reversed the upregulation of the serum malondialdehyde concentration caused by H9N2 AIV infection at 5 dpi and 12 dpi. (c) Baicalin reversed the downregulation of the serum glutathione peroxidase concentration caused by H9N2 AIV infection at 5 dpi and 12 dpi. (d) Baicalin reversed the downregulation of the serum total superoxide dismutase concentration caused by H9N2 AIV infection at 5 dpi and 12 dpi. Data are presented as the mean ± standarddeviation of three independent biological experiments. The differences between groups were analyzed using ANOVA. ^∗^*P* < 0.05 and ^∗∗^*P* < 0.01.

Oxidative stress is a negative effect of free radicals in the body and is considered to be an important factor leading to inflammation [14]. The serum antioxidant capacity and total superoxide dismutase, glutathione peroxidase, and malondialdehyde concentrations are important serum antioxidant indicators. Therefore, we tested the above four indexes in the serum. The results show that at 5 dpi and 12 dpi, the total antioxidant capacity, total superoxide dismutase activity, and glutathione peroxidase activity were significantly decreased in the infected group. However, in the baicalin plus H9N2 AIV infection group, the contents of the above three indexes were significantly higher than in the infected group (*P* < 0.01 and *P* < 0.05) (Figures 5(a), 5(c), and 5(d)). On the contrary, the results show that at 5 dpi and 12 dpi, the serum malondialdehyde concentration significantly increased in the infected group. When baicalin was fed to infected chickens, the content of serum malondialdehyde was significantly lower than in the infected group (*P* < 0.01) (Figure 5(b)). These results indicated that the addition of baicalin could alleviate the oxidative stress caused by H9N2 AIV infection in chickens.

### 3.6. The Beneficial Effects of Baicalin on the Health Status of Chickens

**Figure 6:**
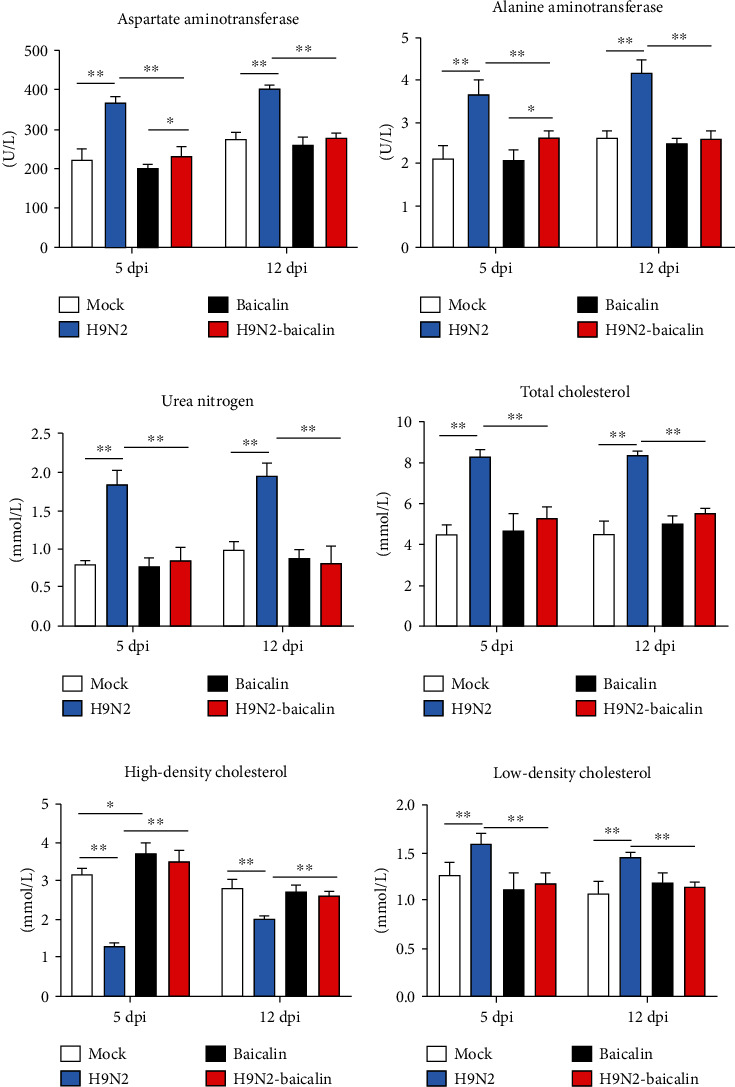
The beneficial effects of baicalin on the health status of chickens. (a) Baicalin reversed the increase in serum aspartate aminotransferase caused by H9N2 AIV infection at 5 dpi and 12 dpi. (b) Baicalin reversed the increase in serum alanine aminotransferase caused by H9N2 AIV infection at 5 dpi and 12 dpi. (c) Baicalin reversed the increase in serum urea nitrogen caused by H9N2 AIV infection at 5 dpi and 12 dpi. (d) Baicalin reversed the increase in serum total cholesterol caused by H9N2 AIV infection at 5 dpi and 12 dpi. (e) Baicalin reversed the decrease in serum high-density cholesterol caused by H9N2 AIV infection at 5 dpi and 12 dpi. (f) Baicalin reversed the increase in serum low-density cholesterol caused by H9N2 AIV infection at 5 dpi and 12 dpi. Data are presented as the mean ± standarddeviation of three independent biological experiments. The differences between groups were analyzed using ANOVA. ^∗^*P* < 0.05 and ^∗∗^*P* < 0.01.

Studies have shown that the occurrence of diseases in the systemic organs of the body can be observed in the blood to a certain extent [23]. Therefore, the detection of blood biochemical indexes is an important method used to judge the occurrence of diseases. The aspartate aminotransferase, alanine aminotransferase, urea nitrogen, total cholesterol, low-density cholesterol, and high-density cholesterol are key blood biochemical indexes that could reflect the health of body. Our study shows that the activity of aspartate aminotransferase and alanine aminotransferase and the content of urea nitrogen in the serum of the infected group were significantly higher than in the control group at 5 dpi and 12 dpi (*P* < 0.01) (Figures 6(a)–6(c)). However, when baicalin was fed to infected chickens, the activity of aspartate aminotransferase and alanine aminotransferase and the content of urea nitrogen in the serum significantly decreased compared with in the infected group (*P* < 0.01) (Figures 6(a)–6(c)), indicating that the addition of baicalin could reduce the activity of aspartate aminotransferase and alanine aminotransferase and decrease the content of urea nitrogen in serum when chickens were infected with H9N2 AIV to avoid damage to the chickens.

Interestingly, our results showed that the addition of baicalin could significantly upregulate high-density cholesterol compared with the mock group (*P* < 0.05) (Figure 6(e)). Besides, at 5 dpi and 12 dpi, the contents of serum total cholesterol and low-density cholesterol significantly increased, while the content of high-density cholesterol significantly decreased in the infected group. However, when baicalin was fed to infected chickens, the total cholesterol and low-density cholesterol contents significantly decreased, while the content of high-density cholesterol significantly increased (*P* < 0.01) (Figures 6(d)–6(f)). Those results indicate that the addition of baicalin can increase the content of high-density cholesterol to enhance the health status of chickens and also can affect the contents of total cholesterol, low-density cholesterol, and high-density cholesterol in serum so as to protect chickens from the damage caused by H9N2 AIV.

### 3.7. Baicalin Alleviated the Inflammatory Response to SPF Chickens by Affecting the mRNA Expression of the Cytokines IFN-r, TNF-*α*, IL-22, IL-17A, IL-6, and IL-1*β*

**Figure 7:**
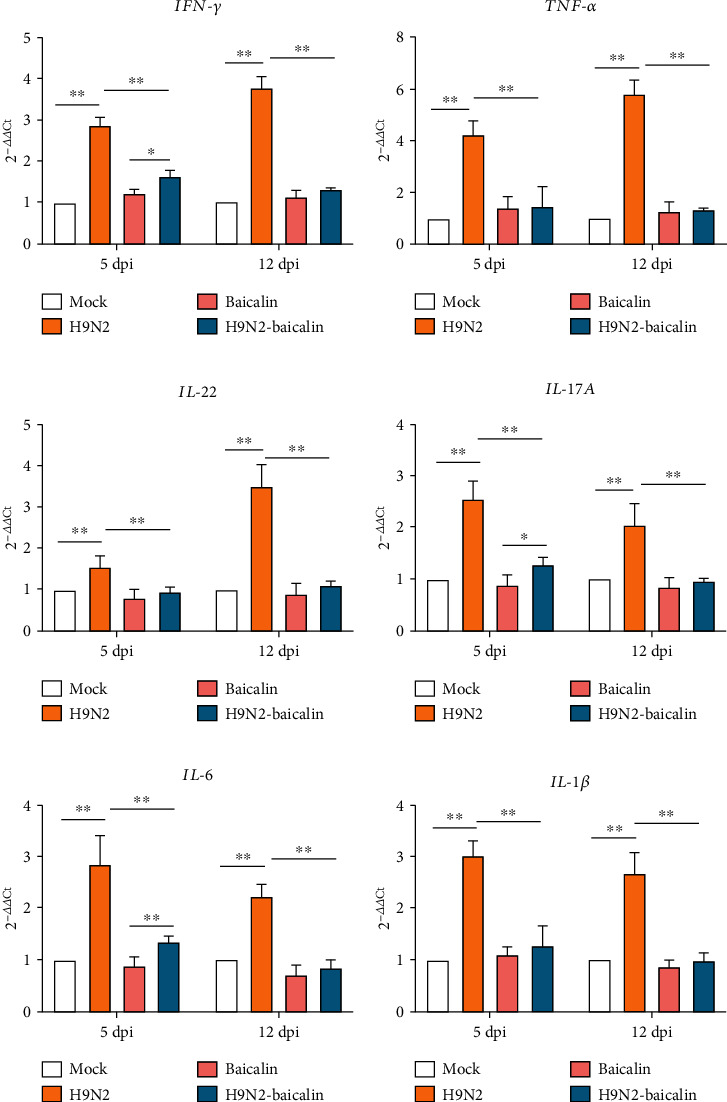
The addition of baicalin alleviated the inflammatory response of SPF chickens by affecting the mRNA expression of the cytokines *IFN-γ*, *TFN-a*, *IL-22*, *IL-17A*, *IL-6*, and *IL-1β*. (a) The mRNA expression of *IFN-γ* at 5 dpi and 12 dpi in different groups. (b) The mRNA expression of *TFN-a* at 5 dpi and 12 dpi in different groups. (c) The mRNA expression of *IL-22* at 5 dpi and 12 dpi in different groups. (d) The mRNA expression of *IL-17A* at 5 dpi and 12 dpi in different groups. (e) The mRNA expression of *IL-6* at 5 dpi and 12 dpi in different groups. (f) The mRNA expression of *IL-1β* at 5 dpi and 12 dpi in different groups. Data are presented as the mean ± standarddeviation of three independent biological experiments. The differences between groups were analyzed using ANOVA. ^∗^*P* < 0.05 and ^∗∗^*P* < 0.01.

Inflammation in body tissues is accompanied by changes in proinflammatory cytokines [15]. *IFN-r*, *TNF-α*, *IL-22*, *IL-17A*, *IL-6*, and *IL-1β* are important proinflammatory cytokines. The purpose of this study was to investigate whether H9N2 AIV infection promotes changes in the above proinflammatory factors in intestinal epithelial cells, and then to explore whether baicalin can inhibit changes to proinflammatory factors caused by H9N2 AIV. As shown in Figures 7(a)–7(f), the mRNA expression levels of *IFN-γ*, *TNF-α*, *IL-22*, *IL-17A*, *IL-6*, and *IL-1β* were significantly upregulated in the infected group at 5 dpi and 12 dpi compared with mock-infected chickens. Furthermore, the addition of baicalin to the infected group significantly decreased the mRNA expression of *IFN-γ*, *TNF-α*, *IL-22*, *IL-17A*, *IL-6*, and *IL-1β* (*P* < 0.01). These results demonstrated that the addition of baicalin could effectively inhibit inflammation of the chicken intestine caused by H9N2 AIV infection.

## 4. Discussion

H9N2 AIV is widely distributed, and the harm it causes is long-lasting and difficult to control. In recent years, secondary bacterial infections caused by H9N2 AIV have been reported from time to time [11, 13, 24]. H9N2 AIV not only poses a threat to public health safety but also leads to serious economic losses for the poultry industry. In recent years, it has been reported that the intestinal microbial community plays a very important role in maintaining host health [25, 26]. Because our previous studies have shown that H9N2 AIV infection can cause poultry intestinal microbiota disorders, it is of great significance to explore new drugs that can effectively regulate intestinal flora to inhibit harmful bacteria caused by H9N2 AIV infection. Moreover, it has not been reported whether Chinese herbal medicine regulates intestinal microorganisms to inhibit the harmful bacteria caused by H9N2 AIV infection. Therefore, this study has important research value and significance.

The intestinal microecological flora of poultry plays an indispensable role in disease control and immune regulation. According to the relationship between the intestinal tract and host, intestinal microbes can be divided into intestinal symbiotic bacteria, conditional pathogenic bacteria, and pathogenic bacteria. The concentration of conditioned pathogens, mainly facultative anaerobes such as *Escherichia coli*, in the intestinal tract is low. When intestinal homeostasis is broken, for example, due to viral infection, the proliferation of conditional pathogenic bacteria is promoted, causing intestinal disorders. In this study, we confirmed that H9N2 AIV infection significantly promotes the proliferation of endogenous *Enterobacteriaceae*, which is consistent with our previous results [11]. Furthermore, the addition of baicalin can inhibit the abundance of *Escherichia*, restore the microecological environment destroyed by H9N2 AIV, and restore *Lactobacillus* as the dominant flora at 5 and 12 dpi, illustrating that baicalin can effectively inhibit the increase in *Escherichia* caused by H9N2 AIV infection and restore the number of beneficial bacteria in the intestinal tract. In the intestinal cavity, bacteria and viruses can penetrate the intestinal mucosa and enter other tissues, organs, and circulatory systems, resulting in bacterial translocation, leading to disease [27]. We confirmed that baicalin can effectively inhibit the translocation of intestinal flora caused by H9N2 AIV infection, illustrating that baicalin can inhibit the translocation of bacteria to the body, thereby inhibiting the bacterial secondary infection caused by H9N2 AIV.

The main pathological manifestations of H9N2 infection are enlargement of the intestinal lymph nodes and necrosis of epithelial cells in the ileum and cecum [28, 29]. The histopathological sections of ileum and cecum in this study also prove that H9N2 AIV infection caused some structural damage to the ileum and cecum. Importantly, baicalin could inhibit intestinal structure injury to the ileum and cecum caused by H9N2 AIV infection. Tight junction proteins of intestinal epithelial cells play an important role in the intestinal mucosal barrier, and damage to them will lead to an increase in cell-to-cell permeability [30]. The proteins and molecules that make up tight junctions mainly include three kinds of transmembrane proteins, including the closure protein (Claudins) and closed small cycle protein (ZOs) [31, 32]. Studies have reported that the occurrence of a variety of intestinal diseases is accompanied by injury to the tight junctions of intestinal epithelial cells and destruction of intestinal barrier structure and function, resulting in pathological changes [33]. In this study, we confirmed that baicalin could inhibit damage to the intestinal mucosal barrier caused by H9N2 AIV infection.

Oxidative stress is involved in several acute and chronic pathological processes, such as acute and chronic kidney disease and neurodegenerative diseases [34]. In addition, oxidative stress can cause intestinal oxidative damage and intestinal mucosal barrier damage [35]. In the antioxidant system of the body, glutathione peroxidase plays an important role in scavenging excess free radicals and restoring normal cell metabolism [36]. The classic method used to evaluate antioxidant capacity is to analyze the levels of some specific antioxidant components, such as total superoxide dismutase and glutathione peroxidase. Total antioxidant capacity is an important indicator that reflects the total antioxidant capacity in the body [37]. A previous study showed that baicalin suppresses diet and nonalcoholic steatohepatitis by inhibiting the JNK signaling pathway and suppressing inflammation and oxidative stress [38]. Hence, it is worth investigating whether baicalin inhibits inflammation caused by H9N2 AIV through affecting oxidative stress indexes. Interestingly, in this study, we confirmed that baicalin can alleviate oxidative stress caused by H9N2 AIV infection and inhibit inflammation.

Blood has the function of maintaining the stability of the environment in the body, and the serum biochemical index reflects the physiological state and health status of the animal body, which can be used as a standard to measure the health of animals [23]. Increases in aspartate aminotransferase and alanine aminotransferase indicate liver injury, while an increase in urea nitrogen indicates kidney injury [39–41]. Low-density lipoprotein is a kind of lipoprotein particle that carries cholesterol into peripheral tissue cells, and an increase in low-density lipoprotein will seriously affect the function of the body. The concentration of high-density cholesterol reflects the quantity of high-density lipoproteins in the plasma, and the main function of this compound is to transport excess cholesterol from extrahepatic tissues to the liver to prevent excess cholesterol accumulation in these tissues [42, 43]. In this study, we confirmed that the addition of baicalin to infected chickens can reduce the concentrations of urea nitrogen, aspartate aminotransferase, alanine aminotransferase, total cholesterol, and low-density cholesterol, while it can increase the concentration of high-density cholesterol in the serum, illustrating that baicalin could protect the body from damage caused by H9N2 AIV infection. Cytokines play a very important role in the immune response. During the occurrence of disease, the expression of proinflammatory cytokines in the body is abnormal, resulting in low immune function or pathological damage [44, 45]. Our results show that the relative transcription levels of *IL-17A, IL-22, IL-6, IL-1β, IFN-γ*, and *TNF-α* increased at 5 and 12 days post H9N2 AIV infection, and these results are consistent with our previous research results and other reports [11, 46]. Furthermore, the addition of baicalin in the infection group was able to alleviate the inflammation caused by H9N2 AIV in chickens. These results once again prove that baicalin can effectively inhibit the inflammation caused by H9N2 AIV infection. To sum up, baicalin can effectively treat bacterial secondary infection and inflammation caused by H9N2 AIV infection.

This study proves, for the first time, that baicalin can effectively inhibit the intestinal microbial disorder caused by H9N2 AIV infection and promote the replacement of *Escherichia* by intestinal beneficial bacteria. In addition, baicalin can inhibit the translocation of intestinal bacteria caused by H9N2 AIV infection, thereby inhibiting secondary infection. Furthermore, baicalin has the effect of minimizing intestinal structure injury, inhibiting damage to ileal mucus layer construction and tight junctions as well as injury to SPF chickens' post-H9N2 AIV infection by affecting blood biochemical indexes, improving the antioxidant capacity, and inhibiting inflammatory cytokines, alleviating the inflammatory response of SPF chickens caused by H9N2 AIV infection. This study provides a new theoretical basis for clinical prevention and control of secondary bacterial infection and inflammation caused by H9N2 AIV infection.

## Figures and Tables

**Table 1:** The design of experimental groups in this study.

Group	Treatment	Number of chickens
Mock group	Basal diet	20
Baicalin group	Basal diet and baicalin	20
H9N2 virus infection group	Basal diet, challenge at 9 days old (3-fold 10^6^ EID_50_/0.1 mL)	20
H9N2 infection and baicalin group	Basal diet and baicalin, challenge at 9 days old (3-fold 10^6^ EID_50_/0.1 mL)	20

**Table 2:** Ingredient composition and nutrient content of the basal diet (%, as-fed basis) [21].

Ingredient	Proportion (kg)	Nutrient levels	Content
Corn	59.00	Metabolic energy/(MJ/kg)	12.64
46% soybean	29.50	Crude protein	21.30
Soybean oil IV	2.80	Calcium	0.83
Corn gluten meal	4.50	Available phosphorus	0.34
Calcium hydrogen phosphate	1.30	Lysine	1.15
Limestone	1.20	Methionine	0.47
Sodium chloride	0.30		
L-Lysine	0.25		
Methionine	0.15		
^1^Premix	1.00		
Total	100		

^1^Premix is provided per kilogram of diet: Mn (MnSO_4_·H_2_O) 60 mg; Fe (FeSO_4_·H_2_O) 66.5 mg; Zn (ZnSO_4_·7H_2_O) 88 mg; Cu (CuSO_4_·5H_2_O) 8.8 mg; I (CaI_2_) 0.7 mg; Se (Na_2_SeO_3_) 0.288 mg; VA11 500 IU; VD33 500 IU; VE 30 mg; VK 33 mg; VB_1_ 3.38 mg; VB_2_ 9.00 mg; VB_6_ 8.96 mg; VB_12_ 0.025 mg; choline chloride 800 mg; calcium pantothenate 13 mg; niacin 45 mg; biotin 0.08 mg; folic acid 1.20 mg.

**Table 3:** Primers for RT-qPCR detection of intestinal inflammation-related cytokines.

Gene names	Primers
*IFN-γ*	F: ATCATACTGAGCCAGATTGTTTCG
	R: TCTTTCACCTTCTTCACGCCAT
*IL-22*	F: CAGGAATCGCACCTACACCT
	R: TCATGTAGCAGCGGTTGTTC
*TFN-α*	F: AGATGGGAAGGGAATGAACC
	R: TCAGAGCATCAACGCAAAAG
*IL-17A*	F: CCATTCCAGGTGCGTGAACT
	R: TTTCTTCTCCAGGCGGTACG
*IL-1β*	F: TGCCTGCAGAAGAAGCCTCG
	R: CTCCGCAGCAGTTTGGTCAT
*IL-6*	F: CGAGGAGAAATGCCTGACGA
	R: TGGGATGACCACTTCATCGG
*GAPDH*	F: AGGCTGAGAACGGGAAACTTG
	R: CACCTGCATCTGCCCATTTG

**Table 4:** Primers for RT-qPCR detection of mucosal barrier-related indexes.

Gene names	Primers
*MUC2*	F: AATGCTGAGTTCTTGCCTAA
	R: GTTGCAGTTCATATCCTGGT
*ZO-1*	F: GCCTGAATCAAACCCAGCAA
	R: TATGCGGCGGTAAGGATGAT
*Claudin-3*	F: GAAGGGCTGTGGATGAACTG
	R: GAGACGATGGTGATCTTGGC
*TFF2*	F: TGGTCCCCAGGACTCAG
	R: GGTAGCACAGTTCACTCGG
*GAPDH*	F: AGGCTGAGAACGGGAAACTTG
	R: CACCTGCATCTGCCCATTTG

**Table 5:** Isolation of bacteria from lung, liver, and mesentery post-H9N2 AIV infection in SPF chickens.

Days postinfection	Tissue	Mock	H9N2	Baicalin-H9N2	Baicalin
5 dpi	Intestinal cavity	+(3/3)	+(3/3)	+(3/3)	+(3/3)
	Liver	-(3/3)	+(1/3)	-(3/3)	-(3/3)
	Lung	-(3/3)	+(2/3)	-(3/3)	-(3/3)
	Mesentery	-(3/3)	+(3/3)	-(3/3)	-(3/3)
12 dpi	Intestinal cavity	+(3/3)	+(3/3)	+(3/3)	+(3/3)
	Liver	-(3/3)	+(3/3)	-(3/3)	-(3/3)
	Lung	-(3/3)	+(3/3)	-(3/3)	-(3/3)
	Mesentery	-(3/3)	+(3/3)	-(3/3)	-(3/3)

+ means isolated bacteria was positive; - means isolated bacteria was negative. Number (front)/number (back): number (front) means the number of positive or negative isolated bacteria; number (back) means the number of total isolated bacteria.

**Table 6:** Identification of Neongreen-tagged bacteria in different tissues of different groups after the infected chickens were drenched with labeled bacteria at 12, 24, 36, and 48 hours.

	Neongreen				H9N2-Neongreen				Baicalin-Neongreen				H9N2-baicalin-Neongreen			
	12 h	24 h	36 h	48 h	12 h	24 h	36 h	48 h	12 h	24 h	36 h	48 h	12 h	24 h	36 h	48 h
Intestinal cavity	+3/3	+3/3	+3/3	+3/3	+3/3	+3/3	+3/3	+3/3	+3/3	+3/3	+3/3	+3/3	+3/3	+3/3	+3/3	+3/3
Mesentery	-3/3	-3/3	-3/3	-3/3	-3/3	+3/3	+2/3	+3/3	-3/3	-3/3	-3/3	-3/3	-3/3	-3/3	-3/3	-3/3
Lung	-3/3	-3/3	-3/3	-3/3	-3/3	+1/3	+2/3	+3/3	-3/3	-3/3	-3/3	-3/3	-3/3	-3/3	-3/3	-3/3
Liver	-3/3	-3/3	-3/3	-3/3	-3/3	-3/3	+1/3	+2/3	-3/3	-3/3	-3/3	-3/3	-3/3	-3/3	-3/3	-3/3

+ means isolated bacteria was positive; - means isolated bacteria was negative. Number (front)/number (back): number (front) means the number of positive or negative isolated bacteria; number (back) means the number of total isolated bacteria.

## Data Availability

The data and materials in the current study are available from the corresponding author on reasonable request.
